# Permeability of the windows of the brain: feasibility of dynamic contrast-enhanced MRI of the circumventricular organs

**DOI:** 10.1186/s12987-020-00228-x

**Published:** 2020-10-28

**Authors:** Inge C. M. Verheggen, Joost J. A. de Jong, Martin P. J. van Boxtel, Alida A. Postma, Frans R. J. Verhey, Jacobus F. A. Jansen, Walter H. Backes

**Affiliations:** 1 Department of Psychiatry and Neuropsychology, Maastricht University, P.O. Box 616, 6200 MD Maastricht, The Netherlands; 2 School for Mental Health and Neuroscience (MHeNs), Maastricht University, Maastricht, The Netherlands; 3 Alzheimer Center Limburg, Maastricht, The Netherlands; 4 Department of Radiology and Nuclear Medicine, Maastricht University Medical Center, Maastricht, The Netherlands; 5 Department of Electrical Engineering, Eindhoven University of Technology, Eindhoven, The Netherlands; 6 School for Cardiovascular Research Institute Maastricht (CARIM), Maastricht University, Maastricht, The Netherlands

**Keywords:** Circumventricular organs, Dynamic contrast-enhanced magnetic resonance imaging, Permeability, Pharmacokinetic modeling

## Abstract

**Background:**

Circumventricular organs (CVOs) are small structures without a blood–brain barrier surrounding the brain ventricles that serve homeostasic functions and facilitate communication between the blood, cerebrospinal fluid and brain. Secretory CVOs release peptides and sensory CVOs regulate signal transmission. However, pathogens may enter the brain through the CVOs and trigger neuroinflammation and neurodegeneration. We investigated the feasibility of dynamic contrast-enhanced (DCE) MRI to assess the CVO permeability characteristics in vivo, and expected significant contrast uptake in these regions, due to blood–brain barrier absence.

**Methods:**

Twenty healthy, middle-aged to older males underwent brain DCE MRI. Pharmacokinetic modeling was applied to contrast concentration time-courses of CVOs, and in reference to white and gray matter. We investigated whether a significant and positive transfer from blood to brain could be measured in the CVOs, and whether this differed between secretory and sensory CVOs or from normal-appearing brain matter.

**Results:**

In both the secretory and sensory CVOs, the transfer constants were significantly positive, and all secretory CVOs had significantly higher transfer than each sensory CVO. The transfer constants in both the secretory and sensory CVOs were higher than in the white and gray matter.

**Conclusions:**

Current measurements confirm the often-held assumption of highly permeable CVOs, of which the secretory types have the strongest blood-to-brain transfer. The current study suggests that DCE MRI could be a promising technique to further assess the function of the CVOs and how pathogens can potentially enter the brain via these structures.

*Trial registration:* Netherlands Trial Register number: NL6358, date of registration: 2017-03-24

## Background

### The circumventricular organs

**Fig. 1:**
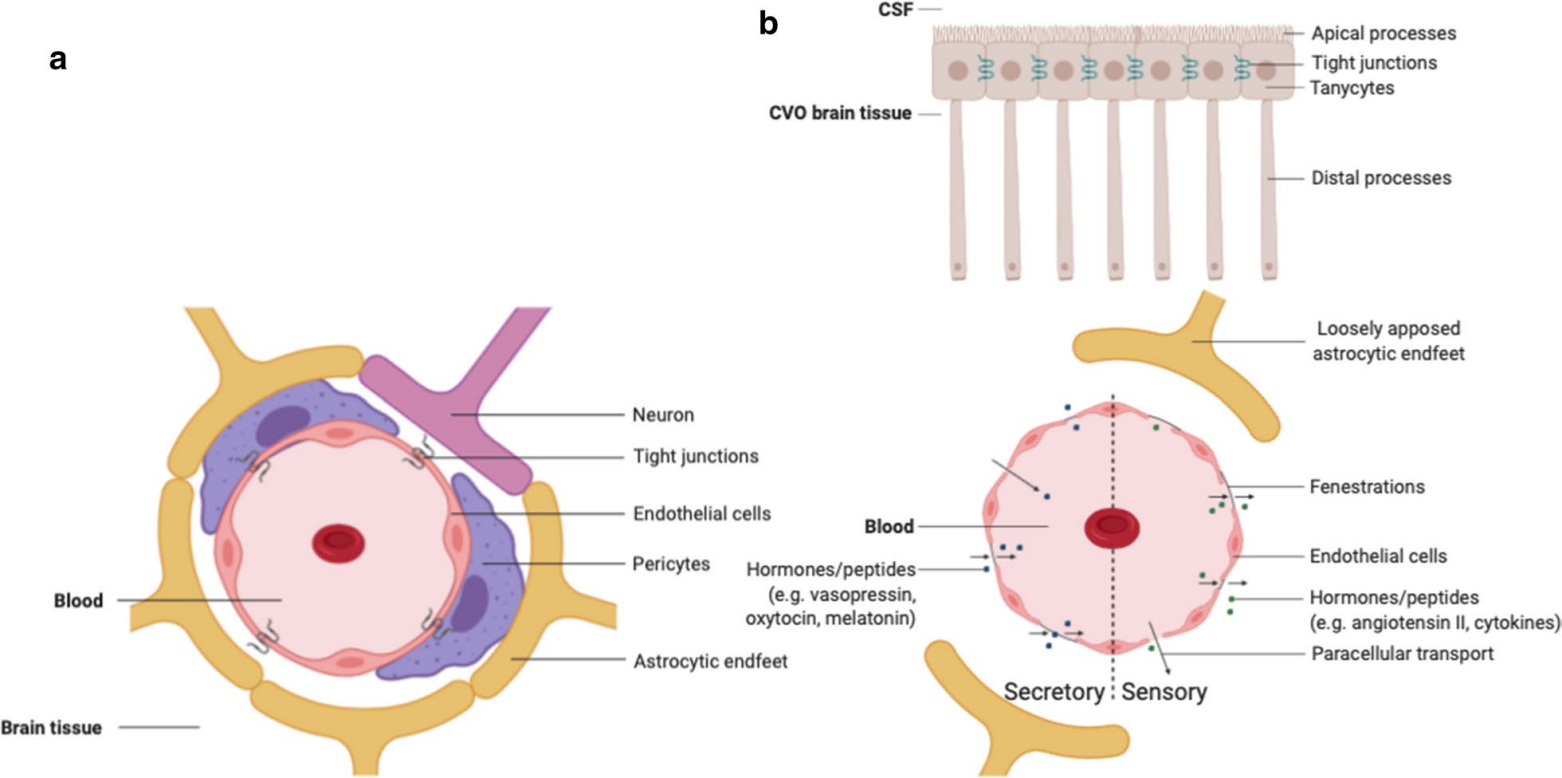
**a** Cross-section of a blood vessel with a normal blood–brain barrier. **b** Circumventricular organ with a cross-section of a capillary with a fenestrated endothelium, with on the left half the secretory and on the right half the sensory type. Tanycytes monitor cerebrospinal fluid composition and pass this information to the circumventricular organ. Figure created with BioRender.com

The circumventricular organs (CVOs) are structures located adjacent to the third and fourth ventricles of the brain [1–3]. An important characteristic of these structures is that they have extensive and highly permeable capillaries that lack a blood–brain barrier (BBB). The vessels in the CVOs branch into a network of fenestrated capillaries with loosely connected astrocytic endfeet (Fig. 1). As a consequence, substances can travel freely between the blood and the CVO tissue, making neurons more susceptible to peripheral signals [4]. The CVOs contain ependymal cells called tanycytes that have apical processes that extend into the cerebrospinal fluid (CSF) to monitor CSF composition and distal processes that contact the fenestrated capillaries to control the exchange of substances between the blood and CSF [1, 4–6]. The tanycytes contain tight junction proteins (Fig. 1). Although a barrier between the vasculature and brain tissue is missing, these tight junctions form a barrier guarding the ventricular components and controlling the diffusion of blood-borne substances to the CSF [7]. The CVOs are considered as points of communication between the blood, CSF and brain parenchyma and, in general, serve to maintain homeostasis [6, 8].

### Functions of the circumventricular organs

The CVOs can be divided in two groups: secretory and sensory types (Fig. 1). The secretory CVOs include the neurohypophysis (NH), median eminence (ME) and pineal gland (PG) [1, 5]. The secretory CVOs are involved in hormone and peptide secretion, neurochemical transport and chemoreception [5] (Table 1). The sensory CVOs include the subfornical organ (SFO), organum vasculosum of the lamina terminalis (OVLT) and area postrema (AP) [5].

**Table 1:** Short overview of the characteristics of the circumventricular organs

	Type	Location	Size [mm]	Primary function	Hormones	Ref.
NH	Secretory	Posterior part of the hypophysis originating from the floor of the third ventricle	1.0–2.9	Releasing hormones received from the paraventricular and supraoptic nuclei	Vasopressin Oxytocin	[2, 23]
ME	Secretory	Extension of the floor of the third ventricle	0.3	Hypophysial portal system: transporting hormones to the hypophysis	Vasopressin Oxytocin	[2, 24]
PG	Secretory	Posterior wall of the third ventricle	1.7	Regulating circadian rhythms	Melatonin	[2, 6]
OVLT	Sensory	Rostral wall of the third ventricle	0.2	Water and sodium homeostasis and immune response	Angiotensin II Cytokines	[2, 4, 6]
SFO	Sensory	Inferior surface of the fornix/roof of the third ventricle	0.3–0.6	Water and sodium homeostasis	Angiotensin II	[2, 6]
AP	Sensory	Floor of the fourth ventricle	0.5	Opening the central canal, cardiovascular and respiratory regulation and controlling the vomiting center	Substance P	[2, 4, 6]

*NH* neurohypophysis, *ME* median eminence, *PG* pineal gland, *SFO* subfornical organ, *OVLT* organum vasculosum of the lamina terminalis, *AP* area postrema

The sensory CVOs can sample molecules from the blood and brain interstitial fluid and pass this information on to other brain structures. They have connections with major neural effector centers for most autonomic functions and have important roles in sodium and water balance, cardiovascular regulation, energy metabolism and immunomodulation [1, 9] (Table 1). The sensory CVOs allow communication between the endocrine, immune and central nervous system and thereby contribute to body fluid homeostasis [4]. For example, angiotensin, a hormone regulating vasoconstriction, arterial pressure and cardiovascular function, acts through signaling into the sensory CVOs [10]. Via their action on CVOs, pharmacotherapy such as angiotensin blockers can decrease blood pressure and cardiovascular morbidity [11].

The subcommissural organ (SCO) is sometimes considered to be a CVO, due to its extensive communication with the CSF [2]. However, the SCO does not have a large network of fenestrated capillaries [5], which is an essential feature of the CVOs, and therefore, we did not include the SCO as a CVO.

The CVOs also play a role in inflammation as they can be entry points for inflammatory cells and are involved in cytokine secretion [12, 13]. The immune system communicates with the brain to activate the hypothalamic–pituitary–adrenal-axis (HPA-axis) [14]. This communication involves activation of the CVOs, which subsequently secrete pro-inflammatory cytokines. Many disorders, such as depression, schizophrenia and Alzheimer’s disease (AD), are associated with disruption of the HPA-axis [15]. Investigating the connection between the CVOs and HPA-circuits may lead to the detection of abnormalities at early stages of disease [4].

Studies in sheep have demonstrated that blood-borne prions can enter the central nervous system (CNS) via the CVOs [16, 17]. Prion accumulation is linked to chronic inflammation in prion disease. Prion accumulation occurs in the CVOs, from where the infection can spread to other brain areas. When pathogens enter through the CVOs, they can activate a cytokine-transcription cascade which produces prostaglandins, subsequently leading to more widespread BBB disruption and CNS pathology [4].

The CVOs have thus been linked to neuroinflammation and characteristics of neurodegenerative disorders and may be involved in neurotoxic protein accumulation. More knowledge on the CVOs could possibly help in the detection of abnormalities at early stages of disease, as they may be the first place for neurotoxins to accumulate, or offer a new pharmaceutical target not hampered by the BBB.

### Imaging techniques for the circumventricular organs

Finding a feasible method to visualize the CVOs is an important first step in gaining more in vivo knowledge on these rather small structures and their role in disease etiology. Since the CVOs do not have a BBB, a contrast agent is expected to transfer rapidly from the blood plasma into the brain tissue. Previous studies have used post-gadolinium enhancement brain MRI to assess CVO visibility [5, 18]. In the current study, we use brain images obtained with a dynamic contrast-enhanced (DCE) MRI technique, which can detect dynamic concentration changes of intravenously administered gadolinium contrast-agent, to investigate whether it is feasible to assess the permeability properties of the CVOs. This DCE MRI technique uses a dual-time resolution protocol, which makes our measurements susceptible to both the early rapid temporal changes capturing the vascular component and the later slower features to capture contrast agent extravasation (i.e. permeability) [19].

### Research question

Our main hypothesis states that significant and positive gadolinium-based contrast agent transfer will be detected in the CVO tissue, due to the absence of the BBB. Additionally, we investigated whether permeability in the secretory CVOs differed from that in the sensory CVOs, and whether permeability in the CVOs differed from the permeability measured in the normal-appearing brain matter. Moreover, cerebral blood flow, which influences blood-to-brain transfer [20, 21], is known to decrease during aging [22]. Therefore, we investigated whether the contrast enhancement in the CVOs was age-dependent.

## Materials and methods

### Participants

Recently, a follow-up on a subsample of participants of the Maastricht Aging Study (MAAS) [25] was conducted, in which 61 individuals who had shown no evidence of cognitive or functional decline were included (Mini-Mental State Examination score ≥ 25; Disability Assessment for Dementia score > 90%; no diagnosis of dementia, mild cognitive impairment or other psychiatric or neurological disorders; no structural brain abnormalities; no cognitive impairment due to substance abuse). All participants provided written informed consent before participation. For the current study, we used the imaging data of the 10 youngest (median age = 53.5 years, age range = 47–56) and the 10 oldest (median age = 73.5 years, age range: 70–91) male participants (Additional file 1: Table 1.1). Previous studies have demonstrated that the estrous cycle influences CVO activity [26–28], so only male individuals were included in this feasibility study.

### MRI acquisition

Anatomical and DCE MRI data were acquired using a 3 Tesla MRI system (Achieva TX, Philips Healthcare, Best, the Netherlands) with a 32-channel head coil. The imaging protocol included a 3D T1-weighted inversion recovery fast gradient echo (repetition time (TR) of 8 ms; inversion time (TI) of 800 ms; echo time (TE) of 4 ms; flip angle of 8°; 1 mm cubic voxel size) for anatomic reference; a 3D T2-weighted fluid attenuation inversion recovery (FLAIR) (TR/TI/TE of 4800/1650/290 ms; flip angle of 90°; 1 mm cubic voxel size) for localizing the CVOs; and a dual-time resolution dynamic contrast-enhanced (DCE) MRI acquisition. The dual-time DCE MRI protocol consisted of two nested pulse sequences, a slow and a fast sequence with a saturation recovery preparation pulse, as described earlier [29]. In short, the fast sequence used a short dynamic scan interval of 3.2 s during the steep signal changes in initial circulations of the contrast agent, while the slow sequence used a longer interval of 30.5 s during the later extravasation phase when the signal changes are much slower. Before contrast administration (pre-contrast), scans of both sequences were acquired. Subsequently, a bolus injection of gadolinium-based contrast agent was performed during the fast sequence (0.1 mmol/kg gadobutrol, Gadavist^**®**^, Bayer AG, Leverkusen, Germany), intravenously in the antecubital vein (injection rate 3 mL/s, 20 mL saline flush). The fast sequence consisted of 29 volumes (TR/TE/delay time (TD) 5.3/2.5/120 ms, voxel size 2 × 2 × 5 mm) and the slow sequence consisted of 30 volumes (TR/TE/TD 5.6/2.5/120 ms, voxel size 1 × 1 × 2 mm). To minimize partial volume, fold over, and inflow effects (of the sagittal sinus superior), an odd number of sagittal orientated slices, 11 for the fast sequence and 75 for the slow sequence, was acquired with the frequency-encoding direction in the craniocaudal direction. T1-mapping with variable delay time settings was performed prior to contrast administration and DCE imaging to enable the conversion of the contrast-enhanced tissue signal intensities to contrast agent concentrations [30].

### Brain regions of interest

**Fig. 2:**
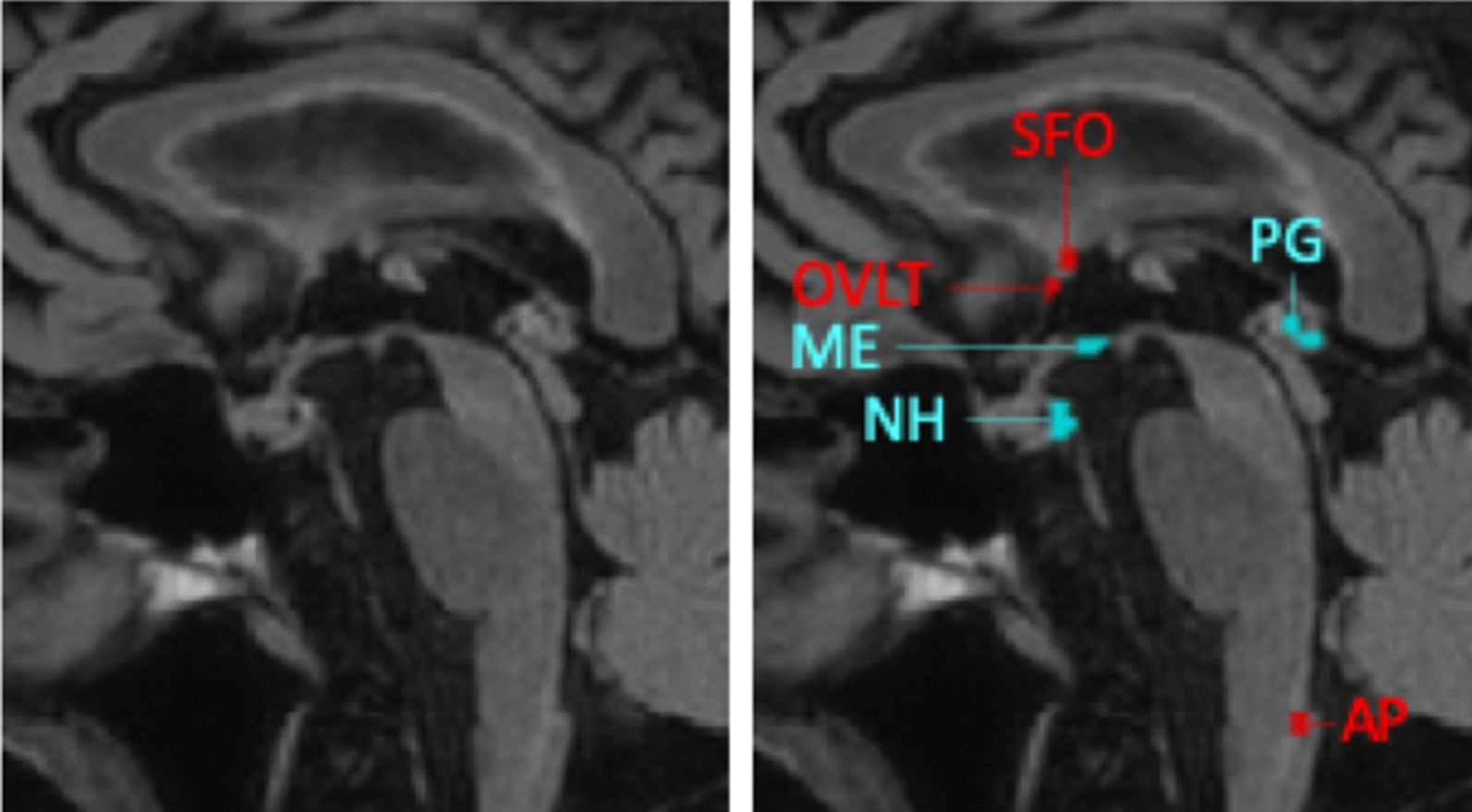
Example of a mid-sagittal anatomical FLAIR image and the regions-of-interest of the secretory (blue) and sensory (red) circumventricular organs. *NH* neurohypophysis, *ME*  median eminence, *PG* pineal gland, *SFO* subfornical organ, *OVLT* organum vasculosum of the lamina terminalis, *AP* area postrema

One researcher (I.C.M.V.) was trained by an experienced neuroradiologist (A.A.P.) in determining the locations of the CVOs on the basis of brain anatomy from the mid-sagittal anatomical FLAIR images. Recognizable anatomical identification marks were used to determine the location of each CVO (e.g. the hypophysis and mammillary body for the NH and ME; the anterior commissure for the OVLT; the hippocampus and fornix for the SFO; the fourth ventricle for the AP), and a limited number of voxels on this exact location was selected, which were saved as region-of-interest (ROI) (Fig. 2).

Additionally, a ROI was placed in the neck muscle, to observe the temporal enhancement time curves for qualitative comparisons of the contrast agent distribution. As quantitative control, the white and gray matter regions were selected, which were segmented using automated software (FreeSurfer, version 6.0.0 [31]). The FreeSurfer segmentation was visually checked by one researcher (I.C.M.V.) with manual adjustments. From the segmented brain regions, the total gray matter (including cortical gray matter, deep gray matter (thalamus, caudate nucleus, putamen, pallidum, amygdala, and accumbens area), and hippocampus) and total white matter volume were extracted [31].

### Pre-processing

The slow and fast dynamic series were motion corrected and spatially aligned using a linear registration procedure with six degrees of freedom (FLIRT, FMRIB’s linear registration tool), with the average pre-contrast slow volume as reference. Next, the motion-corrected dynamic series, the FLAIR images and tissue masks, and T_10_-map were subsequently registered onto the participant’s structural T1-weighted images.

**Fig. 3:**
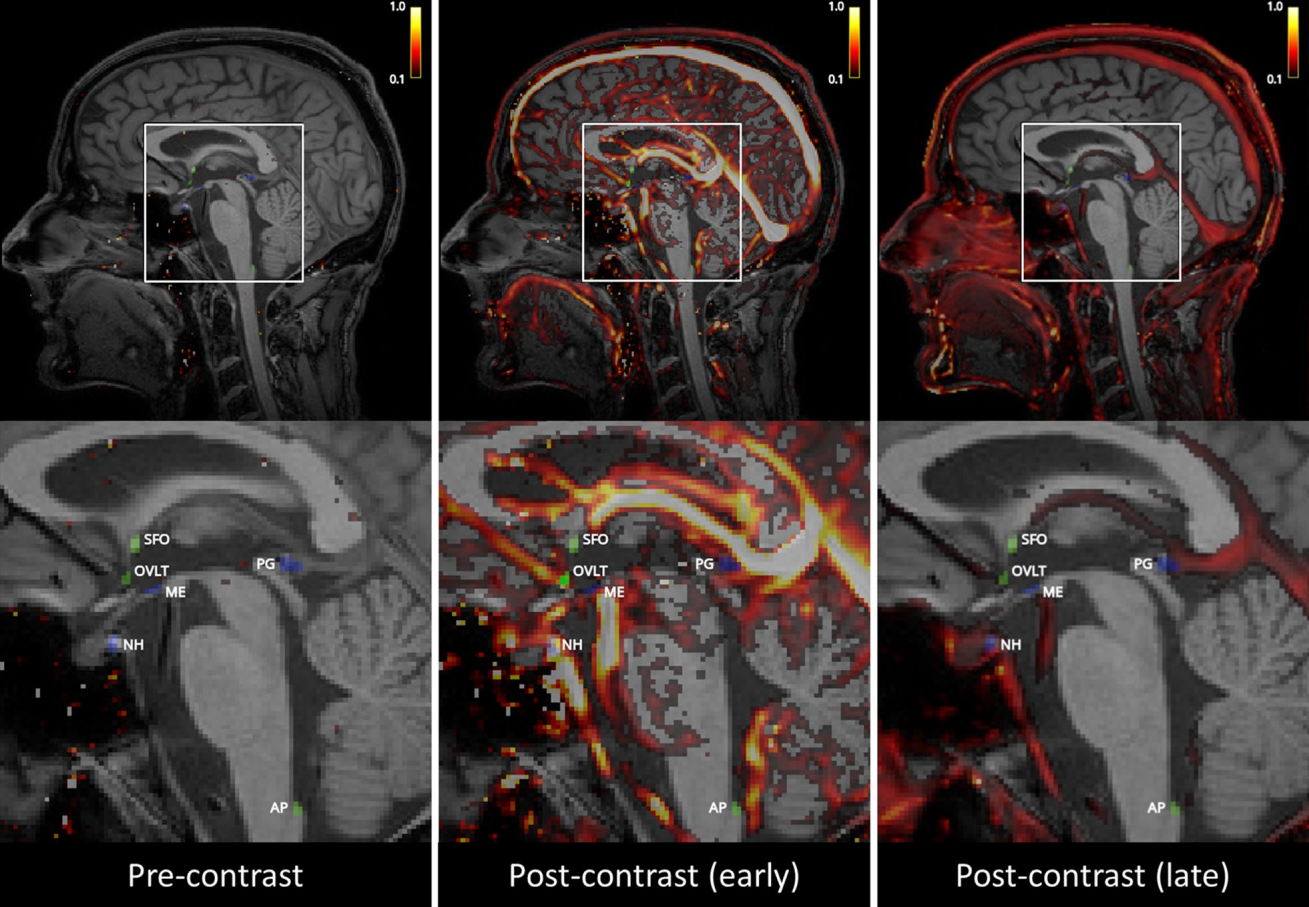
Representative contrast agent concentration maps [mM] showing the distribution of contrast agent before (left), shortly after (middle), and approximately 12 min after (right) contrast agent injection

Individual vascular input functions (VIFs) were extracted from manually (I.C.M.V.) selected voxels (≥ 20) in the superior sagittal sinus [32, 33]. Conversion of MRI signal enhancement to contrast agent concentration was performed differently for the VIF and tissue, and has been described earlier in detail [34]. In short, the VIF signal-to-concentration conversion was implemented using in vitro data (diluted MnCl_2_ stock solution with different gadobutrol concentrations (1–40 mM), baseline T1 relaxation time of 1650 ms, comparable to human blood), whereas the conversion to contrast agent concentration in tissue was performed assuming a linear relationship and a tissue relaxation time calculated from the T_10_-map. Representative contrast agent concentration maps pre- and post-contrast agent injection are shown in Fig. 3. A video of the dynamic contrast agent concentration maps during the whole DCE MRI sequence is included as Additional file 2.

### Pharmacokinetic modeling

**Fig. 4:**
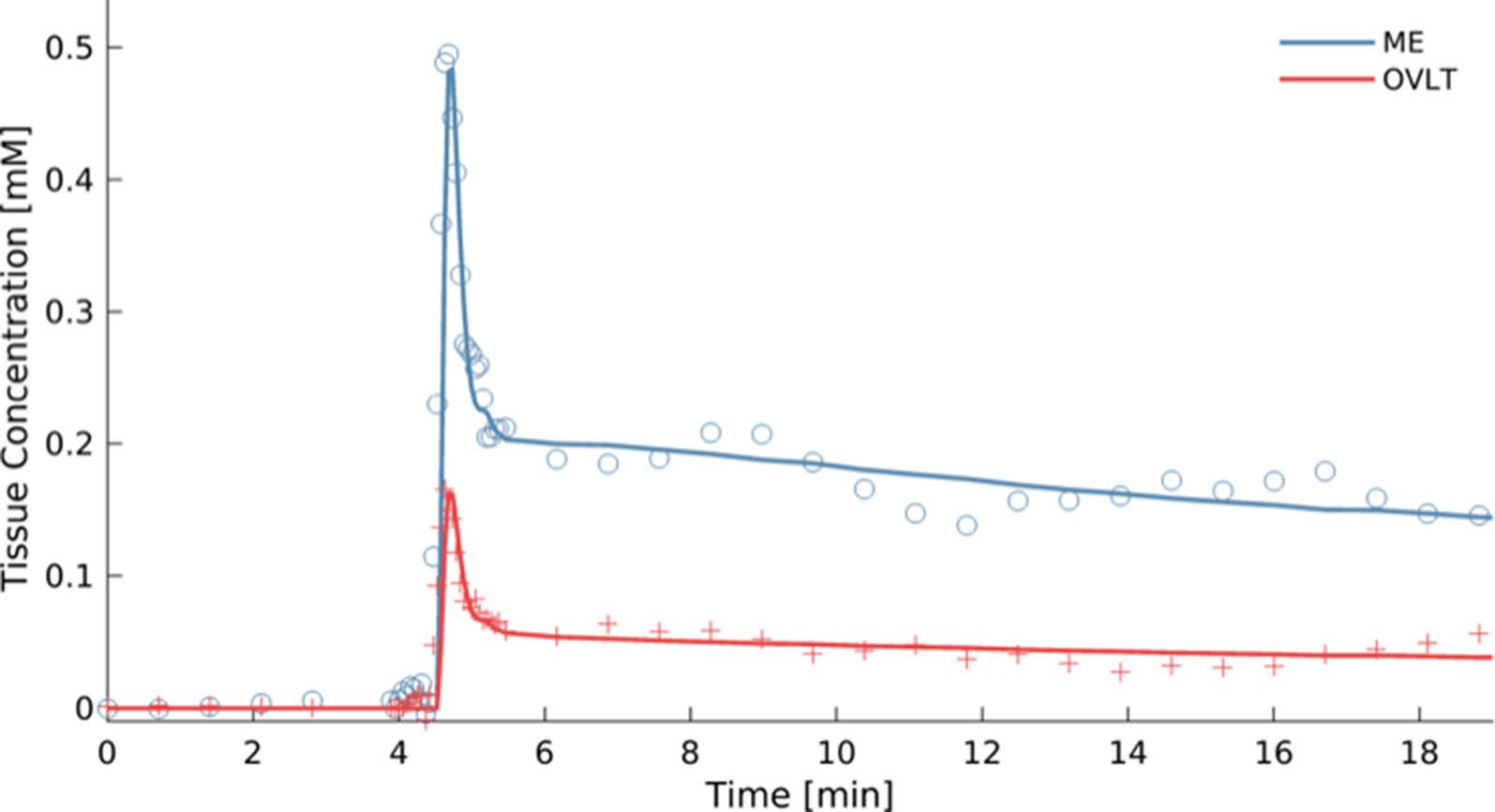
Example of time-concentration curves with good model fits obtained in a secretory (median eminence (ME)) and a sensory (organum vasculosum of the lamina terminalis (OVLT)) circumventricular organ using the extended Tofts model. Note the higher temporal sampling for 1.5 min during the initial steep concentration changes and the slower sampling before and after this period

The CVOs are the points of communication between the blood plasma and the brain tissue and their functionality involves extensive exchange with the blood circulation comprising influx and reflux of solutes [4, 6]. To take both the influx and reflux into account, we applied the extended Tofts model (ETM) to the CVO data, which is a two-compartmental model with a blood compartment and an interstitial compartment with bidirectional transport between these compartments [35]. The CVOs are expected have a high permeability due to the lack of a blood–brain barrier and thus a sufficiently high signal-to-noise ratio. Therefore, the ROI averaged concentration–time data per CVO were fitted using the ETM as implemented in ROCKETSHIP [36] (fitting parameters for K^trans^: starting value of 0.001 min^−1^; lower and upper bound value of -2 and 2 min^−1^; maximum number of iterations of 50; and a function tolerance of 10^−12^) to obtain K^trans^ [min^−1^], the transfer constant from blood plasma to extracellular, extravascular space as measure of permeability, v_p_ [−], the volume fraction of blood plasma within a ROI as measure of perfusion, and v_e_ [−], extravascular space volume fraction as a measure of uptake capacity and retention, for each CVO. The ETM provided sufficiently good fits for both secretory and sensory CVOs (Fig. 4).

Next, to check whether the ETM sufficiently fitted the CVO data, each fit was visually checked (I.C.M.V.) and rated as ‘good’, ‘doutbful’ or ‘bad’, based on how well the modeled curve matched the data points and whether the time-course of the contrast-enhancement curve was sensible (Table 2).

**Table 2:** The median and interquartile range (25th–75th percentile) of the transfer constant (K^trans^) and the number of good fits measured in various regions-of-interest (ROIs)

ROI	ETM K^trans^ [min^−1^]	25th–75th percentile	Number of good fits from a total of 20
Secretory	.22*	.17–.33	19
NH	.40*	.29–.63	19
ME	.048*	.032–.11	19
PG	.078*	.033–.12	16
Sensory	.026*	.021–.036	10
SFO	1.8 · 10^−3^	− .69 · 10^−3^–25·10^−3^	8
OVLT	.019*	.0091–.034	17
AP	10 · 10^−3^*	.87 · 10^−3^– 80·10^−3^	10

	Patlak K^trans^ [min^−1^**]**		
White matter	4.1 · 10^−7^*	.52 · 10^−7^–9.3 · 10^−7^	N.A.^†^
Gray matter	13 · 10^−7^*	5.1 · 10^−7^–17 · 10^−7^	N.A.^†^

*NH* neurohypophysis, *ME* median eminence, *PG* pineal gland, *SFO* subfornical organ, *OVLT* organum vasculosum of the lamina terminalis, *AP* area postrema

* K^trans^ significantly larger than 0 (p < .05)

^†^ Goodness of fit not applicable as a voxel wise Patlak method followed by noise correction using histogram approach was used

The graphical Patlak method was used to calculate the leakage rate (influx) of the contrast agent into the interstitial space of the white and gray matter. This method assumes no reflux from the brain back to the blood and has been demonstrated to be most suitable for assessing pharmacokinetic parameters of normal-appearing brain tissue [37]. Therefore, we applied the graphical Patlak method to assess K^trans^ [min^−1^] and v_p_ [−] in each voxel in the white matter and gray matter. As not in all voxels significant transfer from blood to brain could be measured due to low K^trans^ values in combination with the strong influence of noise, histograms of the K^trans^ values in the white and gray matter were created. These histograms were subsequently corrected for noise [19], after which the mean K^trans^ was calculated as a representative measure for the permeability in the whole white and gray matter region. This approach has been applied previously in healthy controls [19]. For the CVOs, the graphical Patlak method did not appear to be best suitable, as due to their exchange function, reflux must also be taken into account for these structures.

In addition to pharmacokinetic analyses, 1- and 10-min areas under the curve (AUCs) [μM min] were calculated as proxies of gadolinium-based contrast agent wash-in during the circulation phase, and retention during the accumulation phase, respectively. Contrary to the pharmacokinetic parameter, the AUC is not dependent on the type of pharmacokinetic analysis applied and can serve as a data-driven, thus model-free, approach for characterization of contrast enhancement.

### Statistics

To ensure that the transfer constant (K^trans^) of the CVOs gave a realistic representation of the data, the analyses were performed excluding the values obtained from fits that were classified as ‘bad’. Post-hoc, the analyses were repeated also excluding any fits for which it could be doubted whether they were sufficiently good, to see if this would change the results.

Since our participant sample was relatively small and the transfer constants were not normally distributed for each ROI, non-parametric tests were conducted (Part 1: Wilcoxon signed-rank test; Part 2: Mann–Whitney test). All statistical analyses used a level of significance of p < 0.05, and were performed with commercial software (SPSS, version 24.0, IBM Corp., Armonk, NY, USA).

#### Part 1: transfer constants of the CVOs and normal-appearing brain matter

We expected the CVOs to have strong contrast enhancement and significantly positive transfer constants, so a transfer constant significantly higher than 0.

The transfer constants and AUCs were calculated for all CVOs separately, but also for all secretory CVOs combined and all sensory CVOs combined. The Wilcoxon signed-rank test was conducted, comparing the K^trans^ in the secretory and sensory CVOs to a hypothesized median of 0. If a significant result was obtained, post hoc analyses were conducted to see which specific CVOs were significantly different from 0.

As additional analyses, Wilcoxon signed-rank tests were used to compare the K^trans^ between the secretory and sensory CVOs, and between the CVOs and the white and gray matter. Again, a significant result was followed by post hoc analyses to determine which specific CVOs had a significantly different permeability from the other CVOs, or from the white or gray matter.

#### Part 2: age differences

For the second part, we investigate whether the transfer constant (K^trans^) differed between the older and middle-aged group.

For this between-subjects test, the Mann–Whitney test was used and the difference between the older and middle-aged group was assessed within the combined secretory CVOs, combined sensory CVOs, white matter and gray matter. If a significant result was obtained for either the combined secretory or sensory CVOs, post hoc analyses were conducted to see for which specific CVOs the age groups differed most strongly.

## Results

### Concentration curves of different tissue types

**Fig. 5:**
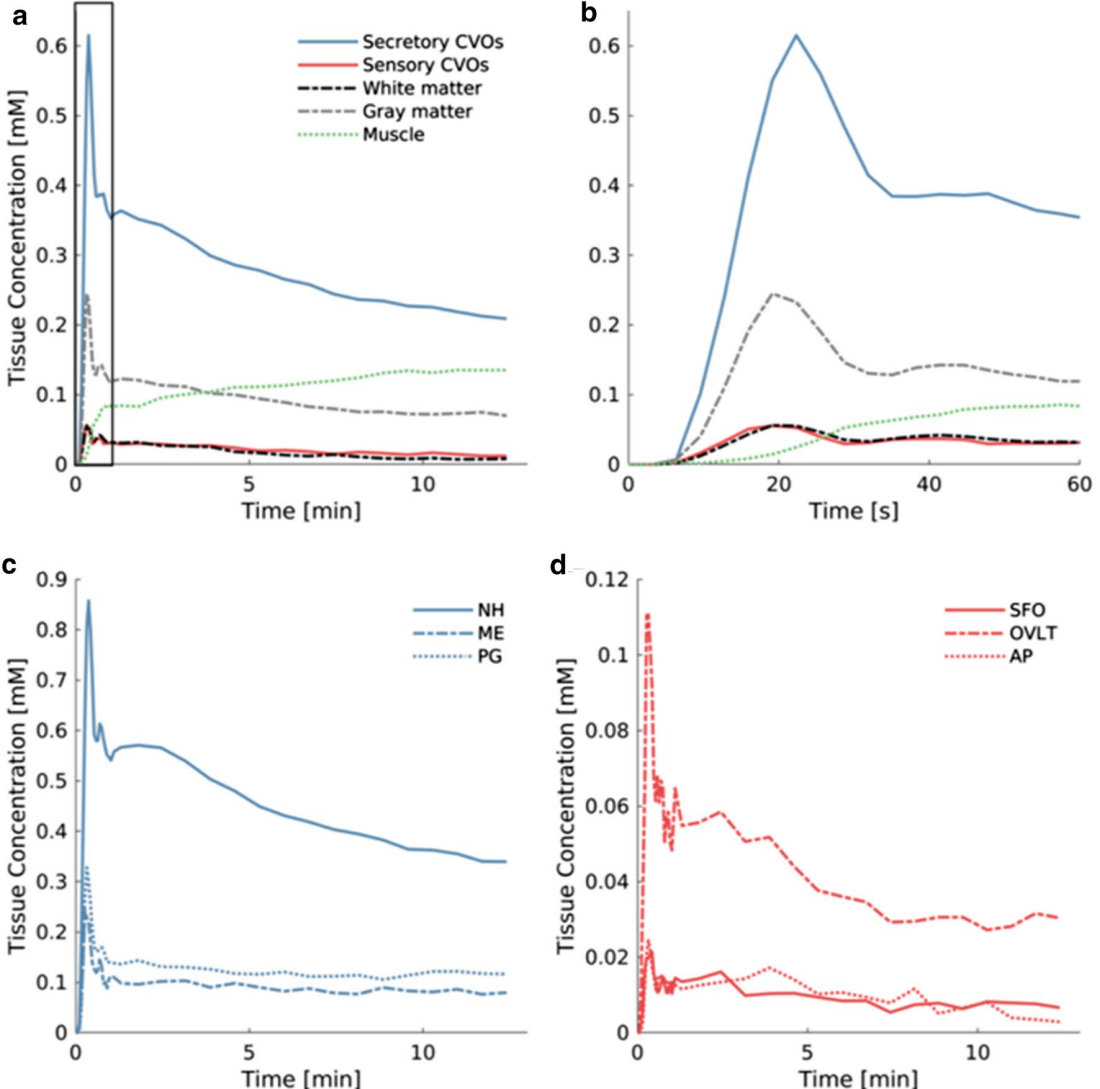
Time course of the gadolinium-based contrast agent concentration in different tissue types and the sagittal sinus (vascular input function (VIF)) averaged over all participants. **a** and **b** In the combined secretory and combined sensory circumventricular organs, white and gray matter and neck muscle since arrival to end of sequence (**a**) and detailed for the first minute (**b**); **c** In the secretory circumventricular organs neurohypophysis (NH), median eminence (ME) and pineal gland (PG); and **d** In the sensory circumventricular organs subfornical organ (SFO), organum vasculosum of the lamina terminalis (OVLT), and area postrema (AP)

We depicted the temporal enhancement profile in CVO tissue and normal-appearing brain matter, as well as muscle tissue (Fig. 5). In the neck muscle tissue, gadolinium-based contrast agent concentration steadily increased (dotted green line). However, the CVO concentration time curves displayed a steep peak followed by gradual decline, which are features also seen in the white and gray matter concentration curves. In this typical pattern, the contrast first quickly circulates through the brain, followed by a slow spread (dispersion). The concentration curves of the secretory CVOs ME and PG showed less steep decline after the initial circulation peaks than the concentration curve of the VIF (Fig. 5c), which is indicative of retention of contrast medium in the CVOs. For the secretory NH structure, retention is also visible by a broadening (hump) after the initial peak (Fig. 5c).

### Transfer constants of the CVOs

For the secretory CVOs, significantly positive transfer constants (K^trans^) were found (p < .001; Table 2). Post-hoc analyses revealed that this was the case for all secretory CVOs (NH, ME and PG; all p values ≤ .001).

Also in the sensory CVOs, significantly positive transfer constants were found (p = .007; Table 2). Post-hoc analyses revealed that this was the case for the OVLT and AP (all p values ≤ .007), but not for the SFO (p = .208). The SFO was also the organ for which the least good fits were obtained (n = 8), with a lower permeability possibly making the data more susceptible to noise and therefore harder to estimate.

Repeating the analyses while also excluding the cases for which the goodness of fit could be doubted, did not change the results.

The values for the blood plasma fraction (v_p_) and the interstitial space fraction (v_e_) can be found in Additional file 3.

### Transfer constants in the secretory CVOs compared to the sensory CVOs

The secretory CVOs had higher transfer constants than the sensory structures (p < .001). Even comparing the secretory CVO with the lowest K^trans^ (ME) to the sensory CVO with the highest K^trans^ (OVLT), revealed that the ME still had a significantly higher transfer constant than the OVLT (p = .014), so all secretory CVOs had significantly higher transfer constants than each sensory CVO.

### Transfer constants in the CVOs compared to the normal-appearing brain matter

Comparing the CVOs to the white and gray matter gave results comparable to the main analyses, with the transfer constants being significantly larger in the CVOs than in the white matter and gray matter (all p values ≤ .004), except for the SFO (both p values = .250).

The model-independent check with AUC values also found stronger contrast enhancement in the CVOs than in the white matter, but only for the secretory structures, which also had the highest permeability parameters when applying the pharmacokinetic analysis. The NH was the only structure that showed significantly stronger contrast enhancement than the gray matter.

Comparing the AUCs of the secretory and sensory CVOs gave the same results as comparing the transfer constants, with the secretory CVOs having significantly stronger contrast enhancement than the sensory CVOs.

These results can be found in Additional file 4.

### Age effect

We found no significant differences in transfer constants between the older and middle-aged group in the secretory CVOs and white matter (all p values ≥ .549). The age effect did approach significance in the sensory CVOs (older: n = 6, middle-aged: n = 4; p = .067), with the older group tending towards a lower permeability, and gray matter (older: n = 10, middle-aged: n = 10; p = .051), with the older group tending towards a higher permeability.

Repeating the analyses while also excluding any fits for which it could be doubted whether they were sufficiently good, gave a significant difference for the sensory CVOs (older: n = 5, middle-aged: n = 4; p = .032), with the older group having significantly lower permeability than the middle-aged group.

## Discussion

### Main findings

In this study, we investigated the feasibility of applying dual-time resolution DCE MRI with a gadolinium-based contrast agent and pharmacokinetic modeling to assess permeability of the CVOs. It was possible to measure gadolinium-based contrast enhancement in these small structures and successfully modeled the temporal uptake curves and derived the influx rate in terms of the transfer constant. Our results demonstrated that positive transfer constants, which were significantly higher than those of the white and gray matter, could be measured in all CVOs, exept for the SFO. Moreover, the transfer constants for the secretory CVOs were significantly higher than for the sensory CVOs. We could not demonstrate any clear significant pharmacokinetic differences in any of the CVOs between the age groups.

### Stronger contrast enhancement in the secretory CVOs

Due to absence of the BBB in the highly fenestrated capillaries, all CVOs were expected to have significantly positive transfer constants, and our results confirmed this hypothesis. The secretory CVOs, and especially the NH as largest peptide-releasing structure, had significantly stronger contrast enhancement and higher transfer constants.

The secretory CVOs being more permeable than the sensory type is supported by previous neurobiological studies. A study in mice previously found higher vascular permeability values in the secretory CVOs relative to the sensory CVOs when using low-molecular-weight contrast agents (fluorescein isothiocyanate (FITC), molecular weight = 0.39 kDa, and Evans Blue, molecular weight = 0.96 kDa), which the researchers attributed to large secretion of peptides into the blood circulation by secretory CVOs [24]. Moreover, a follow-up mice study found evidence that astrocyte-tanycyte connections possibly form an alternative barrier in the sensory CVOs [38]. In the human brain, post-contrast gadolinium studies were conducted to assess CVO visibility [5, 18]. The presence of contrast enhancement in the CVOs on post-contrast T1-weighted 3 Tesla MRI was assessed, as the CVOs were speculated to be mistaken for pathology-related abnormal contrast enhancement [5]. The ME, NH and PG were visible in 100%, 96% and 84% of the cases, respectively. The OVLT was visible in 34% of the cases, while the AP and SFO were hardly ever visualized (2% and 1%, respectively) [5]. A similar study using post-contrast 3D T2-weighted FLAIR 3T MRI images had comparable outcomes [18]. The results of the current study correspond to the results of these previous studies.

The stronger contrast enhancement in the secretory and weaker enhancement in the sensory CVOs found in our study could also have alternative explanations. Blood flow in the sensory CVOs is slower relative to the rest of the brain, for the blood plasma to have better access to the receptors [4]. With slower blood flow, less contrast agent may reach the sensory CVOs.

Another confounding factor could be partial volume effects, due to the small size of the structures compared to the voxel sizes of MRI. The CVOs that seem to have the highest transfer constants, the NH and PG, are also the CVOs in close proximity to large vessels with strong contrast enhancement. Partial volume effects of these large vessels might contribute to the high transfer constants found in these CVOs. The presence of a large vessel in the ROI is reflected by obtaining a higher blood plasma volume (Additional file 3: Table 3.1). The ME, which does not seem to lie close to a large vessel, still has a higher transfer constant than the sensory CVOs. Moreover, an extra check in which the transfer constants were calculated for ROIs with a smiliar proximity to the large vessels, but not containing any CVO tissue, resulted in lower transfer constants compared to the values found for the secretory CVO ROIs. Therefore, while partial volume effects from the large vessels might influence the transfer constants, this demonstrates that these effects cannot fully account for the higher transfer rates found in the secretory CVOs. The sensory CVOs, being smaller than the NH and PG (Table 1 [2]), may experience stronger partial volume effects from the surrounding brain tissue. Due to these partial volume effects, measurements in the sensory CVOs could represent an underestimation. To really understand to what extent partial volume effects from large vessels or surrounding tissue influence the results, more in depth research is necessary. To address the influence of partial volume effects, optimizing the technique with a higher spatial resolution by using ultra-high-field MRI, or applying a more sophisticated pharmacokinetic model controlling for cerebral blood flow in each ROI [39], could be an interesting methodological improvement for future studies.

### Age effect

We expected that the CVOs would have less contrast enhancement in the older group, due to a decrease in blood flow. We did not find a clear significant difference in permeability when comparing the older and middle-aged group. A decrease in blood flow could possibly decrease contrast uptake, but it remains unknown whether blood flow is sufficiently reduced in the older subpopulation to display such an effect. Also, age-related vascular alterations might increase permeability [40] and compensate for the reduced blood flow effect.

The age effect did approach significance in the sensory CVOs (p = .067) and gray matter (p = .051). In the older group, the sensory CVOs tended to be less permeable, suggesting that these structures might be more susceptible to age-related blood flow reduction decreasing permeability. In the gray matter, however, the older group showed a trend towards higher permeability, indicating that this tissue type is more susceptible to age-related vascular alterations increasing permeability, as previous studies have demonstrated [41, 42]. However, it is important to note that the result for the sensory CVOs is based on very small group sizes (older: n = 6, middle-aged: n = 4), and therefore needs further confirmation.

The difficulty to detect a clear age effect could also be because the participants selected from the MAAS study might represent a relatively healthy subsample of the general population, i.e. long-lasting study participants representing ‘survivors’ in an above-average health state. As any age effect is expected to be subtle in such a subsample, it may well be that our study with 10 participants in each group had insufficient power to detect this effect. Especially in the sensory CVOs, exclusion of ‘bad’ fits lead to very small sample sizes, and based on the current study we cannot make any definite claims about the presence of an age effect.

### Additional considerations

We used the ETM to model the CVO time-courses, as, from a theoretical perspective, this model seemed most suitable to the CVOs (blood compartment and interstitial compartment with bidirectional exchange between these compartments [35]), and gave the best fit to our data. However, the ETM can only be used in case of weak vascularization or high perfusion [43, 44]. In tissues with either high vascularization or very slow or very fast exchange, the ETM can still give a good fit to the data, but the derived pharmacokinetic parameters can be unreliable [44]. Therefore, without the certainty that the tissue has either low vascularization or high perfusion, the interpretation of the pharmacokinetic parameters can be misleading. Cerebral tissue is in general highly perfused [45]. For the white and gray matter, for instance, the Patlak method has been established to be the preferred model [37], and this model is also intended for highly perfused tissues [43]. The Patlak method is not suitable for the CVOs though, due to the added assumption of no reflux from the brain back to the blood, which is violated in the CVOs with their exchange function. Therefore, we feel that the application of the ETM to the CVO data was justified [44].

While we applied the ETM to each CVO ROI, an alternative approach would be voxel-by-voxel model selection [46]. According to this approach, when a higher-order model is applied (a model with three or four parameters), this model is routinely tested against lower-order models or plausible alternative models of the same order in each voxel [46, 47]. The four commonly used models in DCE MRI are the Patlak model (K^trans^ and v_p_), the classical Tofts model (TM; K^trans^ and v_e_), the extended Tofts model (ETM; K^trans^, v_p_ and v_e_) and the two-compartmental exchange model (2CXM; K^trans^, v_p_, v_e_ and F_p_ (blood plasma flow)) [43]. As the Patlak model assumes no reflux and the TM is a one-compartment model with negligible blood volume [35], these models are not adequate as they contradict CVO functionality [4, 6]. However, for future studies, it would be interesting to explore the use of voxel-by-voxel model selection and to compare the fits obtained with the ETM and 2CXM, and discern CVO structures from the surrounding tissue based on physiological measures.

We assessed permeability using the gadolinium-based contrast agent gadobutrol. Pathogens, such as prions and amyloid-β, can be found as soluble proteins in the blood [48, 49], but permeability characteristics for these pathogens are not necessarily similar to those of gadobutrol, as the molecular weight of prion proteins (27–30 kDa [50]) or amyloid-β peptides (4 kDa [51]) is higher than the weight of gadobutrol (0.61 kDa [52]). Immunohistochemistry studies in mice have demonstrated that permeability in the CVOs is size-selective, and have found different results with low-molecular weight (< 10 kDa) and high-molecular-weight (≥ 10 kDa) tracers [38, 53, 54]. Studies with high-molecular weight contrast agents should be conducted to confirm these findings in vivo in humans.

## Conclusion

In this study, dual-time resolution DCE MRI was introduced as a possible method to assess CVO permeability. With this method, significantly positive transfer constants could be measured in the CVOs, and the CVOs were shown to have stronger contrast enhancement relative to the normal-appearing brain matter. This observation indicates that current measurements confirm the often-held assumption of highly permeable CVOs, with the secretory CVOs being most permeable. The CVOs are often referred to as ‘windows of the brain’, and pathogens may enter through these windows and trigger an inflammatory response. More advanced ways to assess CVO permeability might eventually help blocking entrance of pathogens, determine factors that disturb homeostasis and possibly contribute to initiatives to overcome neurodegenerative disorders.

## Supplementary information

**Additional file 1.** Participant characteristics.

**Additional file 2.** Video contrast enhancement.

**Additional file 3.** Values for the blood plasma fraction (v_p_) and interstitial space fraction (v_e_).

**Additional file 4.** Contrast enhancement in the CVOs and normal-appearing brain matter.

## Data Availability

The data that support the findings of this study are available on request from the corresponding author [I.C.M.V.]. The data are not publicly available due to their confidential nature.

## Abbreviations

2CXM: Two-compartmental exchange model
AD: Alzheimer’s disease
AP: Area postrema
AUC: Area under the curve
BBB: Blood–brain barrier
CNS: Central nervous system
CSF: Cerebrospinal fluid
CVO: Circumventricular organ
DAD: Disabilities assessment for dementia
DCE MRI: Dynamic contrast-enhanced magnetic resonance imaging
ETM: Extended Tofts model
FITC: Fluorescein isothiocyanate
FLAIR: Fluid attenuated inversion recovery
HPA: Hypothalamic-adrenal–pituitary
MAAS: Maastricht Aging Study
ME: Median eminence
MMSE: Mini-Mental State Examination
NH: Neurohypophysis
NRMSE: Normalized root-mean-square error
NWO: Nederlandse Organisatie voor Wetenschappelijk Onderzoek
OVLT: Organum vasculosum of the lamina terminalis
PG: Pineal gland
ROI: Region of interest
SFO: Subfornical organ
TD: Delay time
TE: Echo time
TI: Inversion time
TM: Tofts model
TR: Repetition time
VIF: Vascular input function
WMA: World Medical Association
WMH: White matter hyperintensity
