# Supplementary material for: Permeability of the windows of the brain: feasibility of dynamic contrast-enhanced MRI of the circumventricular organs
Source: Fluids Barriers CNS. 2020 Oct 28;17:66. doi: 10.1186/s12987-020-00228-x (PMC7594295; doi:10.1186/s12987-020-00228-x)
Supplement: Supplementary file 1 — Additional file 1. Participant characteristics. [file 12987_2020_228_MOESM1_ESM.docx]

**Additional file 1: Participant characteristics**

Table 1.1. Participant characteristics of the two age groups

|  | **Middle-aged** | **Old** |
| --- | --- | --- |
| **N** | 10 | 10 |
| **Age** [years] | 53.5 (50.3 – 55.3) | 73.5 (71.8 – 78.0) |
| **MMSE^a^** | 29.5 (29.0 – 30.0) | 29.0 (27.8 – 30.0) |
| **%WMH Fazekas^b^**  **0/1/2/3** | 10/80/0/10 | 0/70/30/0 |
| **% Diabetes** | 0 | 30 |
| **% Smoker** | 20 | 20 |

Notation: median (25^th^ – 75^th^ percentile)/ percentage

^a^: Mini-Mental State Examination (MMSE (1)): maximum score = 30, cognitively normal ≥ 25

^b^: Fazekas scale with a visual rating score of white matter hyperintensity (WMH) load (2): 0 = absent: none or a single punctuate WMH lesion; 1 = mild: multiple punctuate lesions; 2 = moderate: beginning of confluency of lesions; 3 = severe: large confluent lesions

**References**

1. Folstein MF, Folstein SE, McHugh PR. “Mini-mental state”: a practical method for grading the cognitive state of patients for the clinician. Journal of psychiatric research. 1975;12(3):189-98.

2. Fazekas F, Kleinert R, Offenbacher H, Schmidt R, Kleinert G, Payer F, et al. Pathologic correlates of incidental MRI white matter signal hyperintensities. Neurology. 1993;43(9):1683-.
