# Supplementary material for: Permeability of the windows of the brain: feasibility of dynamic contrast-enhanced MRI of the circumventricular organs
Source: Fluids Barriers CNS. 2020 Oct 28;17:66. doi: 10.1186/s12987-020-00228-x (PMC7594295; doi:10.1186/s12987-020-00228-x)
Supplement: Supplementary file 3 — Additional file 3. Values for the blood plasma fraction (vp) and interstitial space fraction (ve). [file 12987_2020_228_MOESM3_ESM.docx]

**Additional file 3: values for the blood plasma fraction (v_p_) and interstitial space fraction (v_e_)**

Table 3.1. The median and interquartile range (25^th^ – 75^th^ percentile) of the blood plasma fraction (v_p_) and interstitial space fraction (v_e_) measured in various regions-of-interest (ROIs).

| ROI | ETM v_p_  [min^-1^] | 25^th^ – 75^th^ percentile | ETM v_e_  [min^-1^] | 25^th^ – 75^th^ percentile | Number of good fits from a total of 20 |
| --- | --- | --- | --- | --- | --- |
| Secretory | **.11** | .083 – .25 | **.28** | .20 – .32 | **19** |
| NH | .17 | .11 – .33 | .42 | .30 – .53 | 19 |
| ME | .058 | .026 – .094 | .090 | .076 – .13 | 19 |
| PG | .098 | .056 – .13 | .14 | .12 – .19 | 16 |
|  |  |  |  |  |  |
| Sensory | **.022** | .015 – .045 | **.029** | .025 – .045 | **10** |
| SFO | .018 | .006 – .056 | .031 | .019 – 1.0 | 8 |
| OVLT | .030 | .015 – .060 | .037 | .018 – .071 | 17 |
| AP | .040 | .008 – .072 | .050 | .033 – .13 | 11 |
|  |  |  |  |  |  |
|  | **Patlak v_p_**  **[min^-1^]** |  |  |  |  |
| White matter | .010 | .006 – .012 | N.A.^‡^ |  | N.A.^†^ |
| Gray matter | .025 | .019 – .029 | N.A.^‡^ |  | N.A.^†^ |

Abbreviations: NH = neurohypophysis; ME = median eminence; PG = pineal gland; SFO = subfornical organ; OVLT = organum vasculosum of the lamina terminalis; AP = area postrema

^‡^ v_e_ not available as this parameter is not obtained using the Patlak method

^†^ Goodness of fit not applicable as a voxelwise Patlak method followed by noise correction using histrogram approach was used
