# Supplementary material for: Permeability of the windows of the brain: feasibility of dynamic contrast-enhanced MRI of the circumventricular organs
Source: Fluids Barriers CNS. 2020 Oct 28;17:66. doi: 10.1186/s12987-020-00228-x (PMC7594295; doi:10.1186/s12987-020-00228-x)
Supplement: Supplementary file 4 — Additional file 4. Contrast enhancement in the CVOs and normal-appearing brain matter. [file 12987_2020_228_MOESM4_ESM.docx]

**Additional file 4: Contrast enhancement in the CVOs and normal-appearing brain matter**

Table 4.1. Median and interquartile range (25^th^ – 75^th^ percentile) of 1-minute and 10-minute area under the curve (AUC1 and AUC10) measured in various regions-of-interest (ROIs).

| ROI | AUC1  [μM⋅min] | 25^th^ – 75^th^ percentile | AUC10  [μM⋅min] | 25^th^ – 75^th^ percentile |
| --- | --- | --- | --- | --- |
| Secretory | 418^† ‡^ | 260 – 534 | 2947^† ‡^ | 1991 – 3482 |
| NH | 638^†‡^ | 438 – 802 | 4589^†‡^ | 2898 – 5394 |
| ME | 148^†^ | 82 – 230 | 940^†^ | 597 – 1304 |
| PG | 192^†^ | 111 – 270 | 1224^†^ | 763 – 1778 |
| Sensory | 38^‡^ | 4 – 55 | 223^‡^ | 46 – 302 |
| SFO | 17^‡^ | 2 – 50 | 119^‡^ | 34 – 160 |
| OVLT | 69^‡^ | 31 – 113 | 410^‡^ | 207 – 654 |
| AP | 17^‡^ | 0 – 63 | 112^‡^ | 9 – 370 |
| White matter | 41 | 23 – 48 | 176 | 91 – 234 |
| Gray matter | 155 | 77 – 198 | 937 | 469 – 1218 |

† significantly (p<.05) different from white matter, ‡ significantly (p<.05) different from gray matter. Abbreviations: NH = neurohypophysis; ME = median eminence; PG = pineal gland; SFO = subfornical organ; OVLT = organum vasculosum of the lamina terminalis; AP = area postrema; AUC = area under the curve.

For the secretory CVOs, the 1-minute and 10-minute AUCs were significantly larger than for the white and gray matter (Table 4.1., all p-values < .001). Post-hoc analyses demonstrated that all secretory CVOs had significantly higher values than the white matter (all p-values < .001), while the difference with the gray matter was only due to the NH (all p-values < .001).

For the sensory CVOs, the 1-minute and 10-minute AUCs were not significantly different compared to the white matter, and were actually smaller than for the gray matter (Table 4.1., all p-values < .001).

*Age effect*

We found no significant differences between the older and middle-aged group in any of the brain regions (secretory CVOs; sensory CVOs, white matter; gray matter) for the 1-minute or 10-minute AUC (all p-values ≥ .165).
